# The Synthesis and Characterization of Selenium-Doped Bioglass

**DOI:** 10.7759/cureus.61728

**Published:** 2024-06-05

**Authors:** Swetha R, Priyangha P T

**Affiliations:** 1 Department of Periodontology, Saveetha Dental College and Hospitals, Saveetha Institute of Medical and Technical Sciences, Saveetha University, Chennai, IND

**Keywords:** anti-oxidant, anti-inflammatory, selenium, bioglass, amorphous

## Abstract

Background

Bioactive glass, which can form strong bonds with tissues, particularly bones, has become pivotal in tissue engineering. Incorporating biologically active ions like selenium enhances its properties for various biomedical applications, including bone repair and cancer treatment. Selenium's antioxidative properties and role in bone health make it a promising addition to biomaterial.

Aim

The present study was aimed at the preparation and characterization of selenium-doped bioglass.

Materials and methods

Tetraethyl orthosilicate (TEOS) was mixed with ethanol, water, and nitric acid to form a silica network and then supplemented with calcium nitrate, selenium acid sodium nitrate, and orthophosphoric acid. Sequential addition ensured specific functionalities. After sintering at 300 °C for three hours, the viscous solution transformed into powdered selenium-doped bioglass. Characterization involved scanning electron microscope (SEM) for microstructure analysis, attenuated total reflection infrared spectroscopy (ATR-IR) for molecular structure, and X-ray diffraction (XRD) for crystal structure analysis.

Results

SEM analysis of selenium-doped bioglass reveals a uniform distribution of selenium dopants in an amorphous structure, enhancing bioactivity through spherical particles with consistent size, micro-porosity, and roughness, facilitating interactions with biological fluids and tissues. ATR-IR analysis shows peaks corresponding to Si-O-Si and P-O bonds, indicating the presence of phosphate groups essential for biomedical applications within the bioglass network. XRD analysis confirms the amorphous nature of selenium-doped bioglass, with shifts in diffraction peaks confirming selenium incorporation without significant crystallization induction.

Conclusion

The selenium-infused bioglass displays promising versatility due to its amorphous structure, potentially enhancing interactions with biological fluids and tissues. Further research is needed to assess its impact on bone regeneration activity.

## Introduction

In recent years, biomaterials have witnessed a surge in interest, particularly in tissue engineering and regeneration. Among the myriad biomaterials, bioactive glass has emerged as a revolutionary substance, showcasing applications ranging from soft tissue repair to drug delivery [1]. Its clinical utility has also been recognized, marking significant advancements in medical practice. Comprising a three-dimensional silica structure, bioactive glass exhibits the remarkable ability to form robust chemical bonds with tissues, especially bones, upon implantation. Its high biocompatibility and antimicrobial properties make it an ideal candidate for tissue engineering endeavors [2].

Efforts to enhance the biological and physical properties of bioactive glass have led to the incorporation of biologically active ions, resulting in the development of multifunctional biomaterials suited for diverse biomedical applications. These ions, renowned for their benefits to human health, offer a promising alternative to costly pharmaceuticals. Extensive research has explored the biological effects of various metallic dopants, including silver, gallium, zinc, copper, and strontium, highlighting their potential to augment the functionality of bioactive glass [3].

Selenium, an essential micronutrient crucial for human and animal biology, has garnered attention for its antioxidative properties and role in mitigating oxidative stress and inflammation [4]. Notably, selenium-incorporated hydroxyapatite and nanostructured selenium compounds have shown promise as potential biomaterials for bone repair applications [5]. Moreover, selenium compounds have demonstrated inhibitory effects on malignant cell growth, exhibiting selective toxicity toward tumor cells. Such findings underscore the potential of selenium in cancer prevention and treatment [6].

Furthermore, studies have suggested that selenium supplementation may enhance the bone mineral density and improve bone quality, thereby facilitating bone regeneration and fracture healing [7]. Despite these advancements, there remains a dearth of literature on the production of selenium-incorporated bioactive glass. Therefore, the present study aimed to address this gap by focusing on the preparation and characterization of selenium-doped bioglass.

## Materials and methods

Fabrication of selenium-doped bioglass

Tetraethyl orthosilicate (TEOS) was mixed with ethanol and water to form a homogeneous solution. Nitric acid was then added to the prepared homogeneous solution to initiate the formation of the silica network, which was crucial for the material's structure and properties. Sequentially, calcium nitrate, selenium acid sodium nitrate, and orthophosphoric acid were added to the mixture, each contributing specific functionalities to the final material. Calcium nitrate acted as a dopant or stabilizer and played a crucial role in stabilizing the structure, preventing unwanted phase transformations during sintering. Orthophosphoric acid influenced the material's chemical properties and contributed to overall stability and biocompatibility. Selenium acid sodium contributed to the material's unique properties, potentially enhancing its bioactivity. After each addition, water was added to ensure dissolution and homogeneity. The resulting mixture became viscous, resembling a gel-like structure. Subsequently, the viscous solution was sintered at 300 °C for 3 hours to promote the consolidation of the silica network and form the final material. During sintering, the mixture transformed as the heat caused the particles to fuse, forming a more cohesive structure. The resultant selenium-doped bioglass was obtained in powdered form.

Characterization of selenium-doped bioglass

Morphology of Selenium-Doped Bioglass

The microstructure of selenium-doped bioglass was studied using a scanning electron microscope (SEM) (FEI Quanta FEG 650 SEM FEI Company, Hillsboro, Oregon, United States) operated at 2,000 kV, capturing images of the membrane's surface at 500x magnification.

ATR-IR Analysis

Attenuated total reflectance infrared spectroscopy (ATR-IR) was employed to study the molecular structure and chemical composition of the selenium-doped bioglass. The presence of selenium in the bioglass was detected and characterized through changes in the infrared absorption spectrum. Different chemical bonds and functional groups within the bioglass absorbed infrared light at specific wavelengths, leading to characteristic spectrum peaks.

XRD Analysis

X-ray diffraction (XRD) was utilized to analyze the material's crystal structure and phase composition. The resulting diffraction pattern contained peaks corresponding to specific atomic planes within the crystal lattice. By analyzing the positions and intensities of these diffraction peaks, the key characteristics of the material's crystal structure were determined.

## Results

SEM analysis

**Figure 1:**
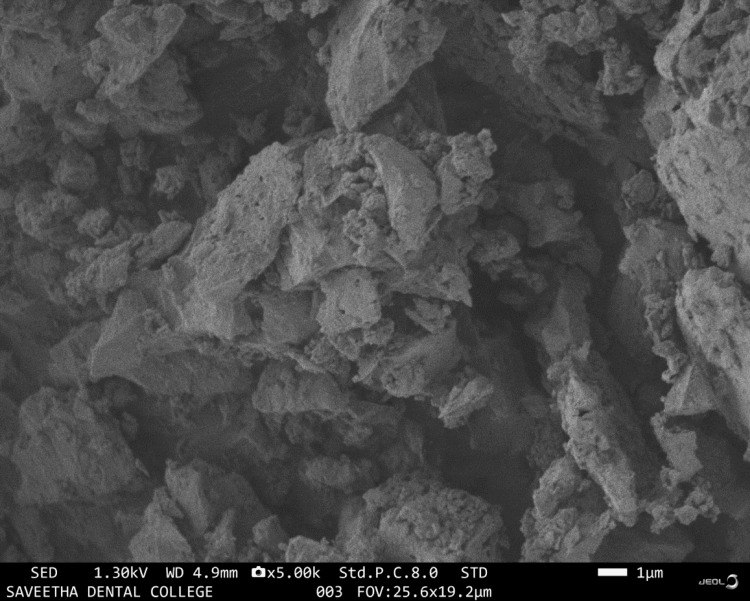
Scanning electron microscopic image of selenium-doped bioglass

The SEM analysis unveils crucial structural insights into selenium-doped bioglass, shedding light on its properties and potential applications. It reveals a uniform distribution of selenium dopants within the bioglass matrix, presenting an amorphous structure rather than a crystalline one as shown in Figure 1. This suggests a random arrangement of atoms, fostering an amorphous or non-crystalline structure. Additionally, the observation of spherical particles with consistent size and shape, along with micro-porosity and roughness, suggests enhanced bioactivity. These features provide an increased surface area for interactions with biological fluids and tissues.

ATR-IR analysis

**Figure 2:**
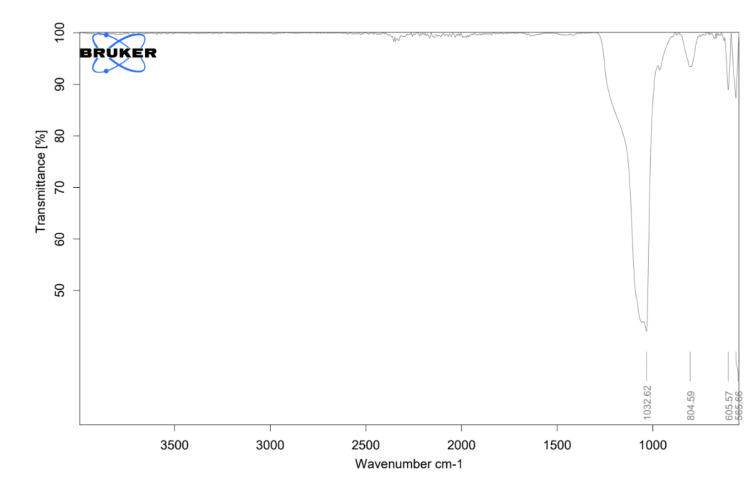
Attenuated total reflectance infrared spectroscopy image analysis of selenium-doped bioglass where the X-axis shows the wavenumber and the Y-axis shows the transmittance

Each peak in the ATR-IR spectrum of selenium-doped bioglass corresponds to specific vibrational modes of chemical bonds present in the material. As depicted in Figure 2, the 1032 cm^-1 peak indicates interactions between phosphorus (P) and oxygen (O) atoms, likely associated with phosphorus-oxygen bonds characteristic of phosphate groups in the bioglass composition. The 804 cm^-1 peak signifies symmetric stretching vibrations of Si-O-Si bonds within the bioglass network, involving simultaneous stretching of silicon-oxygen bonds in a symmetric manner. Vibrational modes associated with phosphorus-oxygen (P-O) bonds are highlighted by the 605 cm^-1 peak, suggesting the presence of phosphate groups essential for biomedical applications. Furthermore, the 565 cm^-1 peak corresponds to bending vibrations of phosphate (P-O) bonds within the bioglass structure, involving angular motion around the bond axis.

XRD analysis

**Figure 3:**
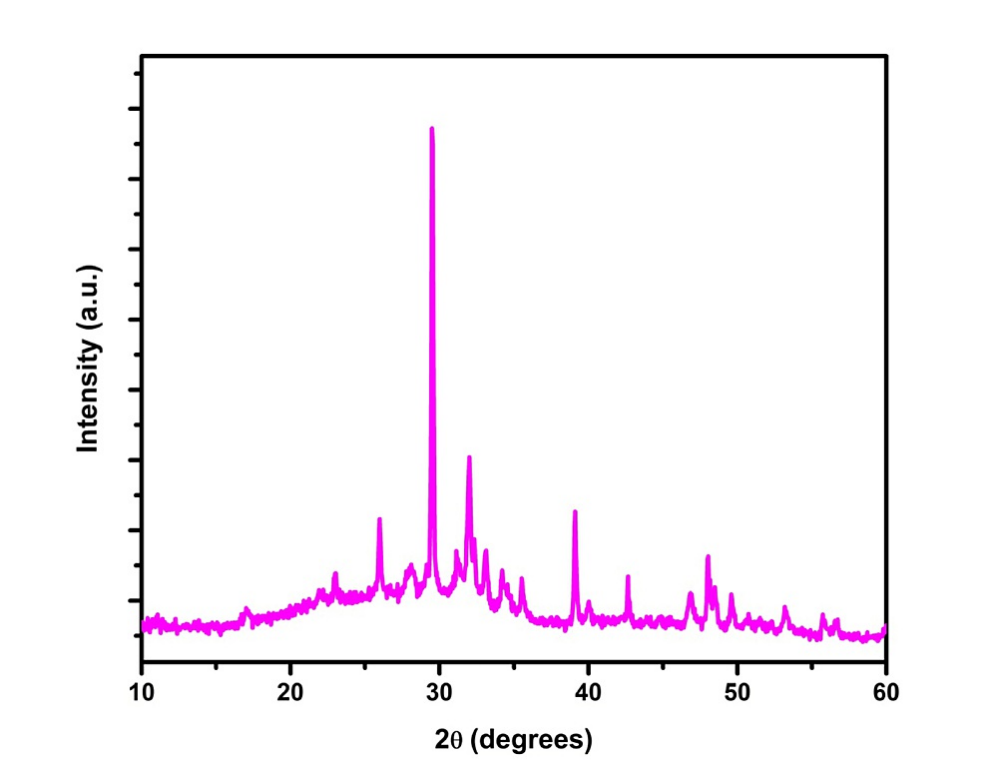
X-ray diffraction image analysis of selenium-doped bioglass in which the X-axis represents the diffraction angle (2θ) and the Y-axis represents the intensity of the diffracted X-rays

The XRD analysis indicates the absence of sharp peaks or diffraction lines, suggesting a primarily amorphous nature in the selenium-doped bioglass as illustrated in Figure 3. The presence of selenium within the bioglass matrix is confirmed by shifts in diffraction peaks and the emergence of new peaks, indicative of its incorporation. The lack of crystalline peaks suggests that selenium doping has not significantly induced crystallization in the glass matrix.

## Discussion

Periodontitis often results in the alveolar bone's support deterioration, detectable through clinical and radiographic evaluation. Achieving successful regeneration of periodontal tissues hinges on respecting the natural biological progression of healing events. While bone autografts are esteemed for their osteogenic properties, they have limitations such as restricted availability, donor site complications, and prolonged operative times [8]. Hence, bone tissue engineering strategies have emerged to tackle these challenges.

Among these approaches, bioglass (BG) has garnered attention in periodontal therapy due to its unique characteristics [9]. BG can generate a highly reactive carbonate hydroxyapatite layer upon contact with bodily fluids, thereby fostering bone regeneration [10]. Bioglass scaffolds offer a three-dimensional framework that supports the proliferation and differentiation of bone cells, facilitating the restoration of damaged or lost bone tissue. Additionally, the dissolution products of bioglass release ions like calcium and phosphorus, further stimulating bone growth and remodeling.

Numerous studies have underscored the benefits of selenium. Incorporating various materials into bioglass to fine-tune its performance has been emphasized in several investigations, highlighting its significance. Hence, in this study, Selenium was utilized as a dopant for bioglass. Selenium was chosen for its antioxidant and anti-inflammatory properties, demonstrating promising synergies when combined with bioglass. In a study, 45S5 bioglass-based scaffolds were coated with selenium nanoparticles and assessed for their antibacterial activity. The results indicated significant antibacterial efficacy against Gram-positive bacteria, including Staphylococcus aureus and Staphylococcus epidermidis [11]. The macrophage metabolism, reactive oxygen species (ROS) scavenging, and bone regeneration potential of selenium-doped mesoporous bioactive glass were examined in another investigation. The findings revealed that the Se-mesoporous bioactive glass (MBG) scaffolds exhibited impressive immunomodulatory properties and robust capabilities for bone regeneration, along with targeted ROS scavenging activity [12]. Furthermore, a separate study explored the drug delivery characteristics of selenium-containing MBG particles. It was observed that these particles demonstrated controlled and sustained release properties, indicating their potential for therapeutic applications [13].

Several studies have investigated the relationship between selenium and bone formation. In one study, selenium nanoparticles were assessed for their effects on osteoblastic differentiation and antimicrobial properties against Porphyromonas gingivalis. The findings indicated positive outcomes, suggesting potential benefits for bone health [14]. Another study evaluated the effectiveness of systemic treatment with alpha-tocopherol and/or sodium selenite in experimental periodontitis. The results concluded that this treatment regimen led to the arrest of disease progression, highlighting its potential therapeutic value [15].

In our study, we subjected the selenium-doped bioglass to in-vitro characterization. SEM analysis unveiled uniformly distributed selenium dopants within an amorphous structure. Additionally, the presence of spherical particles alongside micro-porosity and roughness was noted. These observations suggest that the doped bioglass possesses properties conducive to increasing surface area, thereby enhancing interactions with biological fluids and tissues. Such enhancements could potentially improve biocompatibility and efficacy in biomedical applications. Interestingly, these findings resonate with prior research, particularly a study exploring the influence of carbonated hydroxyapatite and selenium dioxide addition on the mechanical properties of borosilicate inert glass, where analogous SEM observations were made [16].

In ATR-IR analysis, we detected various peaks ranging from 804 cm^-1 to 1032 cm^-1, corresponding to phosphorus-oxygen bonds and stretching vibrations of Si-O-Si bonds, respectively. The significance of observing these peaks lies in the comprehensive insight they provide into the molecular structure of the selenium-doped bioglass. Specifically, identifying peaks associated with phosphorus-oxygen bonds and Si-O-Si bond stretching vibrations offers critical insights into the material's composition and bonding, which are vital for its characterization and potential optimization for biomedical applications.

In XRD analysis, the selenium-doped bioglass exhibited a lack of sharp peaks or diffraction lines, indicating its predominantly amorphous character. This amorphous nature suggests potential improvements in bioactivity and biocompatibility. Moreover, the absence of crystalline peaks suggests that Selenium doping did not trigger unwanted crystallization, thus maintaining the desired glassy structure. Our observations were in line with a study that investigated the impact of selenium inclusion on both the structure and in vitro bioactivity of 45S5 bioglass. The outcomes of this study are congruent with our own findings [17].

Periodontal treatment endeavors to alleviate inflammation and facilitate tissue regeneration efficiently [18]. An ideal bone replacement graft should prompt osteogenesis, cementogenesis, and the formation of a functional periodontal ligament [19]. Bioactive glass, being a ceramic material, is believed to possess bioactive properties that facilitate and encourage osteogenesis, leading to the rapid formation of bone [20]. Studies have indeed shown that incorporating dopants, such as copper, manganese, and zinc, into bioactive glass formulations can enhance their properties [21]. These dopants play crucial roles in influencing the glass's bioactivity, mechanical strength, and biological response [22]. Researchers have investigated the effects of these dopants on the glass's ability to promote bone regeneration and integration with the surrounding tissue, among other factors [23]. When silver is incorporated into bioglass, it fortifies the bioglass against bacterial, fungal, and viral infections, crucial for implants and wound healing [24,25]. Moreover, silver-doped bioglass accelerates the formation of hydroxyapatite, facilitating faster tissue integration and healing [26]. Similarly, copper-doped bioglass demonstrates antimicrobial prowess, fostering a sterile environment around implants while also promoting angiogenesis - the formation of new blood vessels [27]. Recent research has explored incorporating novel dopants like gallium and cobalt into bioglass due to their antibacterial and bone regeneration capabilities [28]. Incorporating gallium into bioglass not only provides structural support but also mitigates the risk of bacterial colonization and has hemostatic properties [29,30]. This approach underscores the versatility of bioactive glasses and their potential for customization to meet specific biomedical needs.

Our study suggests that selenium-doped bioglass holds a potential for periodontal regenerative therapy. Conducting comparative studies with other bone grafts would offer a holistic comprehension of their effectiveness and clinical advantages in real-world applications.

Limitations

Although the doped bioglass demonstrates potential for regeneration in vitro, its clinical application remains to be established. Hence, further in vivo clinical studies are essential to assess the material's clinical effectiveness as a regenerative agent in periodontal therapy.

## Conclusions

The investigation indicates that selenium-infused bioglass exhibited versatility, with its amorphous structure suggesting an increased surface area conducive to interactions with biological fluids and tissues. However, further research is necessary to thoroughly analyze the impact of this bioglass on bone regeneration activity. Building upon the findings of this study, future research efforts can explore innovative strategies, particularly by incorporating various antimicrobial agents into the bioglass to achieve synergistic effects. Additionally, the osteogenic and osteoconductive properties of the formulated bioglass warrant further investigation.
